# The expression and activity of thioredoxin reductase 1 splice variants v1 and v2 regulate the expression of genes associated with differentiation and adhesion

**DOI:** 10.1042/BSR20150236

**Published:** 2015-11-26

**Authors:** Ivan Nalvarte, Anastasios E. Damdimopoulos, Joëlle Rüegg, Giannis Spyrou

**Affiliations:** * Department of Biosciences and Nutrition, Karolinska Institute, SE-141 83 Huddinge, Sweden; † Department of Clinical Neurosciences, Karolinska Institute, SE-171 76 Stockholm, Sweden; ‡ Swedish Toxicology Science Research Center (Swetox), SE-151 36, Södertälje, Sweden; § Department of Clinical and Experimental Medicine, Division of Microbiology and Molecular Medicine, Linköping University, SE-581 85 Linköping, Sweden

**Keywords:** differentiation, migration, oxidative stress, selenium, thioredoxin reductase

## Abstract

The mammalian redox-active selenoprotein thioredoxin reductase (TrxR1) is a main player in redox homoeostasis. It transfers electrons from NADPH to a large variety of substrates, particularly to those containing redox-active cysteines. Previously, we reported that the classical form of cytosolic TrxR1 (TXNRD1_v1), when overexpressed in human embryonic kidney cells (HEK-293), prompted the cells to undergo differentiation [Nalvarte et al. (2004) J. Biol. Chem. **279**, 54510–54517]. In the present study, we show that several genes associated with differentiation and adhesion are differentially expressed in HEK-293 cells stably overexpressing TXNRD1_v1 compared with cells expressing its splice variant TXNRD1_v2. Overexpression of these two splice forms resulted in distinctive effects on various aspects of cellular functions including gene regulation patterns, alteration of growth rate, migration and morphology and susceptibility to selenium-induced toxicity. Furthermore, differentiation of the neuroblastoma cell line SH-SY5Y induced by all-*trans* retinoic acid (ATRA) increased both TXNRD1_v1 and TXNRD1_v2 expressions along with several of the identified genes associated with differentiation and adhesion. Selenium supplementation in the SH-SY5Y cells also induced a differentiated morphology and changed expression of the adhesion protein fibronectin 1 and the differentiation marker cadherin 11, as well as different temporal expression of the studied TXNRD1 variants. These data suggest that both TXNRD1_v1 and TXNRD1_v2 have distinct roles in differentiation, possibly by altering the expression of the genes associated with differentiation, and further emphasize the importance in distinguishing each unique action of different TrxR1 splice forms, especially when studying the gene silencing or knockout of TrxR1.

## Abstract (precis)

Thioredoxin reductase (TrxR1) is involved in redox homoeostasis and cellular differentiation. In the present study, we demonstrate that overexpression of TrxR1 affects genes associated with differentiation and that differentiation increased TrxR1 expression. The TrxR1 splice variant TXNRD1_v2 was also studied in this context.

## INTRODUCTION

The mammalian ubiquitously expressed homodimeric selenoprotein thioredoxin reductase (TrxR) belongs to the nucleotide oxido-reductase family and is a member of the thioredoxin (Trx) system [1–5]. Each homodimer of TrxRs contains a FAD and a NADPH binding motif as well as a penultimate selenocystein (Sec) residue, which makes close contact with the active site (-Cys-Val-Asn-Val-Gly-Cys-) of the adjacent subunit [6–8]. The incorporation of selenium in the active site relies on an intricate translation machinery, and accounts for the major ascribed physiological effects of selenium. Although the main substrate of TrxRs is Trx, the high reactivity of Sec at physiological pH and its accessibility at the C-terminus confer TrxRs broad substrate specificity [9]. TrxRs have been shown to reduce, and thereby activate, several antioxidant proteins and molecules such as glutathione peroxidase (GPx), ubiquinone (Q10), dehydrolipoic acid *S*-nitrosoglutathione (GSNO). Further, they directly reduce hydrogen peroxides and lipid hydroperoxides, selenite, vitamin K and dehydroascorbic acid [4,10,11], hence playing a central role in the antioxidant defence. Apart from the classical 54 kDa cytosolic form of TrxR1 several isomers exist, such as a mitochondrial form (TrxR2) [12,13], a glutathione reductase containing form (TGR) and a glutaredoxin containing form (TrxR-Grx, TXNRD1_v3) where the latter two are mainly expressed in testis [14,15]. In addition, TrxR1 is subject to extensive splicing, primarily at the 5′ end, giving rise to several products both on the mRNA and protein level [16]. To distinguish between the splicing variants the classical TrxR1 will here be designated as TXNRD1_v1.

Previously, we have shown that TXNRD1_v1 overexpression down-regulates cell migration and increases the expression of specific markers associated with epithelial cell differentiation in human embryonic kidney cells (HEK-293) [17,18]. Although the complex mechanisms behind this finding may be difficult to elucidate, the interaction of TXNRD1_v1 with several redox sensitive proteins, including transcription factors, may be a plausible explanation for increased differentiation. In fact, it has previously been shown that nuclear TXNRD1_v1 [19] and TXNRD1_v2 [20] interact with oestrogen receptors (ERs), which belong to the transcription factor family of hormone-activated nuclear receptors (NRs).

To further investigate the involvement of TXNRD1_v1 and TXNRD1_v2 in the expression of differentiation markers in the TXNRD1_v1/v2 overexpressing HEK-293 cells we performed a gene expression profiling analysis of these cells. We observed a striking up-regulation of several genes that are associated with differentiation, development and cell motility in cells overexpressing TXNRD1_v1/v2 compared with control cells carrying the empty plasmid. Although many genes were similarly regulated between the two splice variants some were found oppositely regulated, indicating different functional roles of TXNRD1_v2 ascribed to the N-terminal domain of that protein. To validate these results we induced the human neuroblastoma cell line SH-SY5Y to undergo differentiation using all-*trans*-retinoic acid (ATRA). We show that both ATRA and selenite induced the expression of TXNRD1_v1 and TXNRD1_v2 and this affected the expression of the two adhesion proteins and differentiation markers fibronectin 1 and cadherin 11. These data provide novel insights into the role of both TXNRD1_v1 and TXNRD1_v2 in cell differentiation and adhesion.

## EXPERIMENTAL

### Construction of stable cell lines

We designed primers TR1a-5′ (5′-GAATTCACCACCAT-GGACGGCCCTGAAGATCTTC-3′), TR1a-3′ (5′-CTGA-ATTCGCCAAATGAGATGAGGACG-3′) and TR1b-5′ (5′-GGAATTCACCACCATGTCATGTGAGGACGG-3′) to amplify TXNRD1_v1 and TXNRD1_v2, according to standard PCR procedures and to introduce EcoRI restriction sites. The amplified products were cloned into pGEMTeasy vector (Promega) and sequenced. Stable cell lines overexpressing TXNRD1_v1 or TXNRD1_v2 were generated using the pIRESneo vector system (Clontech). pGEMTeasy/TXNRD1_v1 and pGEMTeasy/TXNRD1_v2 were digested with EcoRI and the inserts were cloned into pIRESneo vectors. Ten micrograms of pIRESneo/TXNRD1_v1 or pIRESneo/TXNRD1_v2 was used to transfect HEK-293 cells (A.T.C.C.) using polyethyleneimine (PEI) (Sigma). 0.1 mg/ml DNA in water were mixed with 0.5 μl of 0.1 M PEI, vortexed and incubated for 10 min at room temperature. The mixture was then added to the HEK-293 cells cultured in a 6-well plate and the cells were allowed to grow for 2 days. The medium was changed to medium supplemented with 1 mg/ml G418 (Calbiochem) and then re-changed to fresh G418-supplemented medium every 2–3 days for 2 weeks. This process selects cells that have stably incorporated the plasmid into their genomic DNA. Resistant clones were picked and transferred to new 6-well plates and cultured extensively with G418-supplemented medium for additional 2 weeks. Thereafter the cells were split into 25 cm^2^-flasks (BD Biosciences) containing medium without G418 supplementation. Different clones were analysed for expression by activity measurements and Western blot analysis. Control cells (HEK-Control) were prepared by transfecting HEK-293 cells with the empty pIRESneo vector to keep the DNA amount constant and were selected as above.

### Cell culturing and differentiation

HEK-293 cells (A.T.C.C.) were cultured in Dulbecco's-modified Eagle's medium (DMEM; containing 1mg/mL glucose) and F12 nutrient mixture (ratio 1:1) supplemented with 10% fetal calf serum (FCS) (all from Life Technologies). The HEK-293 cells were reselected every 4 weeks with 1 mg/ml G418. The SH-SY5Y cells (A.T.C.C.) were grown in DMEM (containing 4.5 mg/ml glucose) and 10% FCS. The HeLa cells (A.T.C.C.) were maintained in DMEM medium (containing 1 mg/ml glucose) supplemented with 2 mM L-glutamine and 10% FCS. All cells were cultured at 37°C and 5% CO_2_ in a humidified incubator. The SH-SY5Y cells were differentiated using a non-toxic concentration of ATRA of 5 μM for 5 days. Where indicated the HEK-293 and SH-SY5Y cells were treated with an effective concentration of 0.2 μM sodium selenite (Se).

### Western blotting

The TXNRD1_v1 and TXNRD1_v2 antibodies were generated by immunizing rabbits with purified inactive TXNRD1_v1 and with a TXNRD1_v2 specific peptide (KQRKIGGHGPTLKAY, Figure 1A) respectively and purified as previously described (20). The cells from 75 cm^2^ culture flasks were trypsinated, pelleted and washed once with ice-cold phosphate buffered saline (PBS), pH 7.4. The cells were freeze thawed once, resuspended in 0.25 M sucrose, 10 mM Tris/base pH 7.2, 2 mM EDTA and 0.1 mM PMSF (Sigma) and homogenized using a tight-fit glass-glass homogenizer. The homogenates were then centrifuged at 25000 ***g***, 4°C for 30 min to remove cellular debris. The extracts were separated on SDS/PAGE, transferred to a nitrocellulose membrane (GE Life Sciences) and probed with TXNRD1_v1 antibody [17], TXNRD1_v2 antibody [20], fibronectin antibody, cadherin antibody or Hsp-90 antibody (all from Sigma). The bound antibodies were visualized using secondary anti-mouse or anti-rabbit horseradish peroxidase linked secondary antibody (GE Life Sciences) and ECL detection kit (GE Life Sciences).

**Figure 1:**
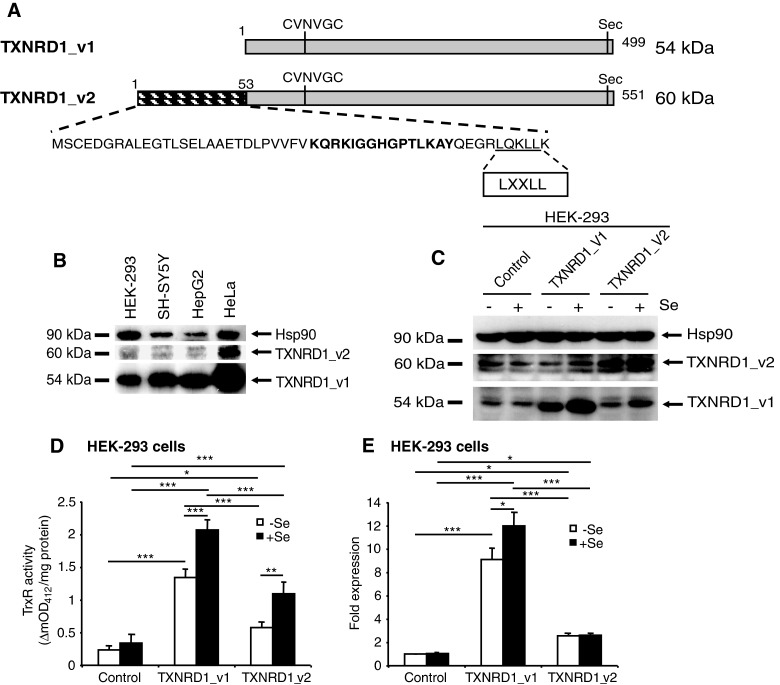
Expression of TXNRD1_v1 and TXNRD1_v2 (**A**) Comparison between TXNRD1_v1 and v2 splicing variants. In contrast with TXNRD1_v1, TXNRD1v_2 has 52 extra N-terminal amino acids, encompassing a LXXLL consensus sequence (NR-Box) (underlined). Peptide sequence used for antibody production is shown in bold. Active sites are represented by CVNVGC motifs. Sec, selenocysteine. (**B**) Western blot analysis of TXNRD1_v1 and TXNRD1_v2 in cell lines. Hsp-90 was used as a loading control. (**C**) Western blot analysis of TXNRD1_v1 and TXNRD1_v2 in HEK-293 cells overexpressing either of the isoforms upon incubation with or without of 0.2 μM Se for 3 days. (**D**) TrxR1 activity and (**E**) real-time qPCR analysis in empty vector control, TXNRD1_v1 or TXNRD1_v2 overexpressing HEK-293 cells with or without 0.2 μM Se treatment for 3 days. Each bar represent mean for at least three independent experiments completed in duplicates and error bars correspond to S.D., *P<0.05, **P<0.01, ***P<0.001 using Student's *t*test.

### TrxR activity measurement

The cell extracts were prepared as above and assayed for total TrxR activity according to the method by Holmgren and Björnstedt [21] with some slight modifications. In brief, triplicates of 50 μg protein extract were added to wells of a 96-well plate (BD-Biosciences) containing 1 M HEPES–NaOH, pH 7.5, 4 mM EDTA, 200 μM NADPH and 1 mg/ml insulin yielding a final volume of 100 μl. The reaction was started by the addition of 10 μM *Escherichia coli* Trx (Promega) to the wells followed by incubation at 37°C for 20 min. Blank samples were treated similarly except for no addition of Trx. The reaction was terminated by the addition of 200 μl 6 M guanidine–HCl in 0.2 M Tris/HCl containing 0.4 mg/ml 5,5′-dithiobis-2-nitrobenzoate (DTNB, Sigma) producing 2-nitro-5-thiobenzoate. The absorbance was read spectrophotometrically at 412 nm (PowerWaveX, Bio-Tek). Where indicated, NADPH oxidation was measured by adding the extracts to the wells of a 96-well UV plate (Nunc) containing 50 mM Tris/HCl, pH 7.4, 1 mM EDTA, 1% BSA and 600 μM fresh NADPH. The reaction was started by the addition of 40 μM Se and followed spectrophotometrically at 340 nm. Blank sample was treated equally but without selenite addition. Blank values were subtracted from each sample.

### Trx1 redox state analysis

The redox state of Trx1 was determined using thiol-trapping with the high molecular mass probe 4-acetamido-4′-maleimidylstilbene-2,2′-disulfonic acid (AMS, Life technologies) followed by Western blot analysis as described previously [22].

### Microarray analysis

HEK-control, HEK-TXNRD1_v1 and HEK-TXNRD1_v2 cells were grown in 25 cm^2^ culture dishes until 80% confluence and then trypsinated and pelleted. The pellets were lysed and RNA was extracted using the RNAeasy RNA-extraction kit (Qiagen). The total RNA quality was tested using the Agilent Bioanalyzer at the Karolinska Institute Bioinformatics and expression analysis core facility and the hybridization proceeded according to the standard Affymetrix protocols (http://www.affymetrix.com/support/technical/manuals.affx) using the Human genome U133A 2.0 array chip (Affymetrix) representing 18400 transcripts and variants including 14500 well characterized human genes. The microarray results were normalized to internal Affymetrix controls using GeneChip Operating Software (GCOS) and with a standard set of R methods according to standardized protocols (Affymetrix).

### Reverse transcriptase-PCR and real-time qPCR

Cells were grown in 25 cm^2^ culture flasks as described above and harvested. The RNA was extracted using the RNAeasy RNA extraction kit (Qiagen) according to the manufacturer's instructions and the RNA quality was tested as described above. Possible genomic DNA was digested by treating 1 μg of RNA with DNase I (Life Technologies) in DNase I reaction buffer for 15 min at room temperature. Then the DNase I was inactivated with 2.2 mM EDTA and samples were incubated at 65°C for 10 min. The cDNA was generated with the First-strand cDNA synthesis system for RT-PCR using SuperScript III reverse transcriptase, dNTPs and random hexamers (all from Life Technologies). By following the manufacturers protocols the RT-products were generated using 1 μg RNA and incubating at 25°C for 5 min, then at 50°C for 45 min followed by 15 min inactivation at 70°C. The real-time PCR was performed using 0.4 μl template and 300 nM individual primer pairs (see Table 1) in 1X Power SYBR Green PCR master mixture (Applied Biosystems) to make up a total volume of 10 μl. The 7500 Fast real-time PCR System (Applied Biosystems) was used to detect amplified target sequences. The primers (Supplementary Table II) were annealed at 60°C for 45 PCR cycles. Experimental values represent at least three different reaction experiments completed in duplicates. The relative mRNA expression was calculated with the ΔΔCt method using 18S rRNA as an internal control, since this gene demonstrated less variability and higher reproducibility.

**Table 1:** Differentially expressed genes in TXNRD1_v1 and TXNRD_v2 overexpressing cells Differential expression of genes associated with differentiation, adhesion, migration and/or tumorigenesis in HEK cells overexpressing TXNRD1_v1 and TXNRD1_v2 compared with HEK-control cells transfected with empty plasmid. In addition, the Se-proteins GPx3 and SELT are included. Real-time qPCR analysis of gene expression is indicated. *Values represent fold changes based on microarray analyses with detection *P* values < 0.0001. No change in gene expression in the microarray data is represented by NC. The data were confirmed using quantitative PCR analysis (real-time qPCR) and the Ct values were compared following normalization to 18S rRNA. Mean ratio of expression and S.E.M. was calculated using ∆∆Ct differences.

			Microarray data*		Real-time qPCR data		Involvement			
			Fold change in expression		Fold change in expression					
Gene name	Symbol	NCBI accession number	TXNRD1_v1	TXNRD1_v2	TXNRD1_v1	TXNRD1_v2	Differentiation/development	Adhesion/migration	Tumorigenesis	Function
*Up-regulated genes*										
Cadherin 11	CDH11	NM_001797	68.6	29.9	303.6±82.5	45.6±16.8		X	X	Tight junction transmembrane protein seen to be overexpressed in several adenocarcinomas.
Galectin 8	LGALS8	NM_006499	26.0	55.7	21.9±2.6	169.2±75.9	X	X		Modulator of cell adhesion. Involved differentiation by regulating cell growth, apoptosis and migration.
Claudin 7	CLDN7	NM_001307	21.1	5.70	30.4±13.7	4.4±1.3	X	X	X	Tight junction formation, overexpressed in several adenocarcinomas.
Jak & microtubule interacting protein	JAKMIP2	NM_014790	8.04	7.52	220.1±161.5	141.4±68.4		X		A non-receptor tyrosine kinase that binds Janus kinases and is involved in the cytokine signalling cascades. It profoundly perturbs the microtubule network and contributes to the cell polarity.
Stratifin	SFN	NM_006142	7.46	NC	12.3±4.6	4.1±0.62			X	Anticancer protein regulated by p53 and is involved in cell-cycle control. Often silenced in tumours.
Fibroblast growth factor 13	FGF13	NM_004114	7.12	8.61	14.9±6.1	6.4±2.6	X	X	X	Signal transduction molecule involved in embryonic development, cell growth, morphogenesis and tumour growth and invasion.
SRY (sex determining region Y)-box 9	SOX9	NM_000346	2.46	2.47	1.2±0.29	1.3±0.56	X		X	Anti-tumorigenic transcription factor involved in gonadal development by decreasing the rate of cellular proliferation and increasing the sensitivity to apoptosis.
Fibronectin 1	FN1	NM_002026	NC	3.72	0.75±0.21	2.9±0.80	X	X	X	A glycoprotein located at the cell surface or extracellular matrix. It is involved in cell adhesion, migration during embryogenesis, blood coagulation and metastasis.
*Down-regulated genes*										
Zn-finger protein 22 (KOX15)	ZNF22	NM_006963	0.015	0.025			X			Transcription factor involved in developmental specificity
Protein kinase, X-linked	PRKX	NM_002760	0.044	0.22	0.57±0.13	1.4±0.67	X	X		A serine/threonine kinase that is thought to be involved in regulation of epithelial morphology during kidney development.
SRY (sex determining region Y)-box 3	SOX3	NM_005634	0.11	0.22	0.05±0.04	10.5×10^−3^±5.3×10^−3^	X		X	Transcription factor involved in the regulation of embryonic development. It may exert different effects depending on its interacting partner and may function in apoptotic pathways as well as in tumorigenesis.
Oestrogen-related receptor γ (ERRγ)	ESRRG	NM_001438	NC	0.019	0.51±0.16	3.2×10^−3^±1.6×10^−3^	X		X	Transcription factor that plays a role in development and differentiation of several tissues. Often seen overexpressed in tumours responsive to hormonal treatment. Binds peptides containing a NR-box.
Glutathione peroxidase 3	GPx3	NM_002084	0.23	NC	0.79±0.24	1.0±0.72				A selenoprotein that protects from oxidative damage by reducing peroxides.
Selenoprotein T	SELT	NM_016275	0.25	0.62	0.67±0.35	0.97±0.38				A selenoprotein. Unknown function.

### Boyden chamber assay

HEK-Control, HEK-TXNRD1_v1 and HEK-TXNRD1_v2 cells were grown for 3 days in 0, 0.2 or 1μM Se and thereafter trypsinized and prepared in single cell suspension. Cells were seeded into collagen type I Biocoat inserts (BD-Biosciences) at 30000 cells in 500 μl of complete medium. Outer wells were filled with 500 μl complete medium. The medium was supplemented with 0, 0.2 or 1 μM Se and the cells were allowed to transmigrate to the outer membrane of the insert for 24 h. Inner medium and cells were carefully removed and the outer cells were fixated in 4% paraformaldehyde and visualized using an Axiovert S100 microscope (Carl Zeiss) following 1% crystal violet staining.

### Statistical methods

Unless stated otherwise, statistical significance was determined using an unpaired, two-tailed Student's *t*test, assuming unequal variances (single comparisons); or a one-way ANOVA followed by the Tukey *post-hoc* test (multiple comparisons). Differences were considered significant if the *P*-value was <0.05, *P<0.05, **P<0.01, ***P<0.001, for all tests.

## RESULTS

### TXNRD1_v1 and TXNRD1_v2 overexpression

Apart from the classical cytosolic form of Trx1, TXNRD1_v1 (*TXNRD_v1* gene product), there exists several splice variants of which one form, containing a 53 amino acid N-terminal extension, is designated TXNRD1_v2 (*TXNRD_v2* gene product, also known as KM-102-derived reductase-like factor, KDRF) (Figure 1A) [16,23,24]. This form contains an N-terminal LXXLL motif, a so-called NR-box, which typically interacts with NRs. To detect TXNRD1_v2 an antibody was generated raised against amino acids 28–42 [20]. Both TXNRD1_v1 and v2 were found ubiquitously expressed in most tissues [20] and we could also verify their endogenous expression in several cell lines with the highest expression in HeLa cells (Figure 1B). To study both TrxR1 isoforms in detail, we created HEK-293 cell stably overexpressing either TXNRD1_v1 (HEK-TXNRD1_v1) or TXNRD1_v2 (HEK-TXNRD1_v2), which showed increased levels of the respective protein compared with control cells transfected with the empty vector (HEK-Control) (Figure 1C). In HEK-293 cells the TXNRD1_v2 antibody detected a slightly lower duplicate band with similar intensity, which is likely to be a post-translated variant of TXNRD1_v2. The full blots are shown in Supplementary Figure S1 to assess antibody cross reactivity and revealed no cross reactivity of TXNRD1_v2 antibody with TXNRD1_v1. However, TXNRD1_v1 antibody seems to cross-react with TXNRD1_v2 (Supplementary Figure 1) and reveals that TXNRD1_v2 levels are generally lower expressed. To reach the full activity of the TrxRs, we pretreated the HEK-293 cells overexpressing the v1 or v2 splice variants with 0.2 μM Se for 3 days [17], on top of the selenium available in normal growth medium (7–40 nM selenium) [25]. This resulted in an increase in TXNRD1_v1 protein levels (Figure 1C), which was also reflected in the TrxR activity in cytosols of TXNRD1_v1/v2 overexpressing HEK-293 cells (Figure 1D) and mRNA expression of TXNRD1_v1 (Figure 1E). Generally TXNRD1_v2 showed significantly lower expression levels than TXNRD1_v1. Selenium supplementation did not alter the expression of this gene (Figures 1C and 1E), but rather rendered it more active (Figure 1D). The gene expression results were confirmed with two additional primer sets for TXNRD1_v2. The primer-binding specificity was confirmed by using the pure pIRESneo/TXNRD1_v2 construct or the pIRESneo/TXNRD1_v1 vector as templates (data not shown). The gene expression analyses suggested that TXNRD1_v2 has a lower expression or a higher turnover of mRNA in HEK-293 cells, in line with the observed lover TXNRD1_v2 protein levels. Furthermore, although TXNRD1_v1 and TXNRD1_v2 are mainly cytoplasmic proteins, they are also found in the nucleus [20]. In our studies we could not see any significant change in nuclear/cytoplasmic localization upon selenium supplementation (data not shown).

### Effect of TXNRD1_v1 and TXNRD1_v2 overexpression on growth and migration

As reported earlier, we found that TXNRD1_v1 overexpression caused HEK-293 cells to grow slower and to have a higher resistance towards selenium-induced toxicity [17,26]. However, although TXNRD1_v2 overexpression also resulted in slower growth, it was not as protective against selenium toxicity as TXNRD1_v1 at 10 μM Se (Figure 2). This could, as above, possibly be explained by the generally lower expression of TXNRD1_v2 and/or poorer incorporation of selenium into the protein.

**Figure 2:**
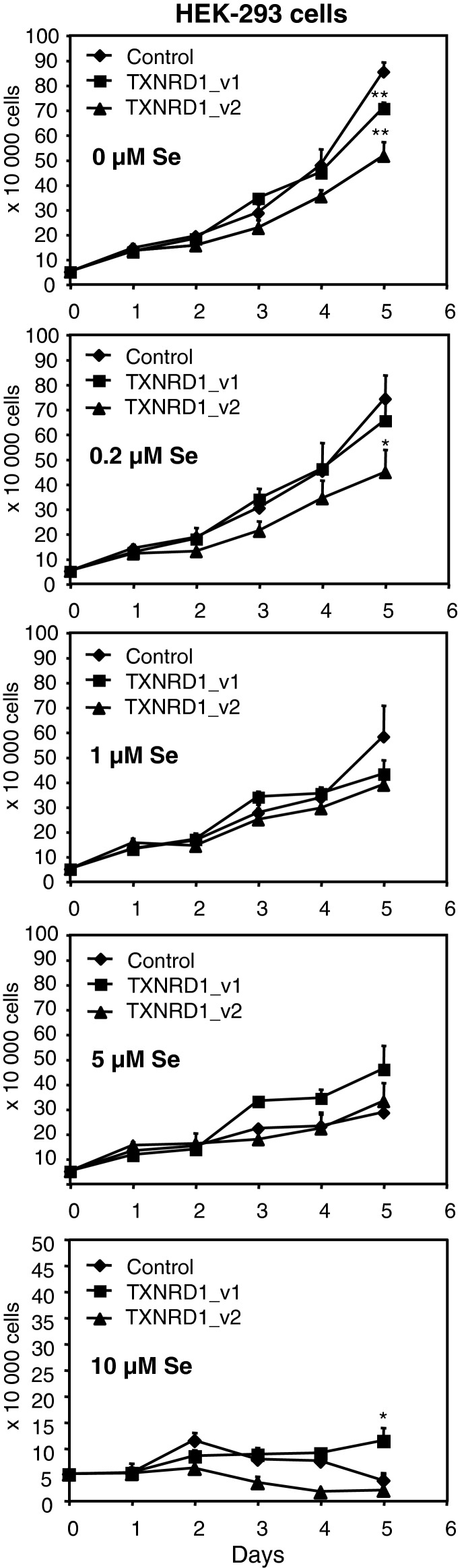
Growth characteristics of HEK-293 cells overexpressing TXNRD1v_1 and TXNRD1_v2 The cells were grown in plain medium, 0.2, 1, 5 or 10 μM selenium-supplemented media for the indicated days. Cells were counted using Trypan Blue exclusion. Error bars correspond to S.E.M. of three biological replicates counted in triplicates, *P<0.05, **P<0.01, ***P<0.001.

A morphologic analysis of HEK-TXNRD1_v1/v2 cells compared with control cells showed aggregation of HEK-TXNRD1_v1 cells in colonies (Figure 3A), suggesting inhibited migration as previously described [18]. HEK-TXNRD1_v2 cells, on the other hand, showed a similar morphologic appearance as control cells (Figure 3A), although with a slightly lower cell count. These observations were confirmed as TXNRD1_v1 and, to a lower extent, TXNRD1_v2 overexpressing cells showed reduced basal migration in Boyden chambers compared with control cells (Figure 3B). Selenium supplementation had no effect on migration of HEK-Control or HEK-TXNRD1_v2 cells. However, 1 μM selenium supplementation further reduced the ability of HEK-TXNRD1_v1 cells to migrate (Figure 3B). The effect of selenium on morphology and migration of HEK-TXNRD1_v1 cells correlates with the increase in TXNRD1_v1 activity and expression (Figure 1D).

**Figure 3:**
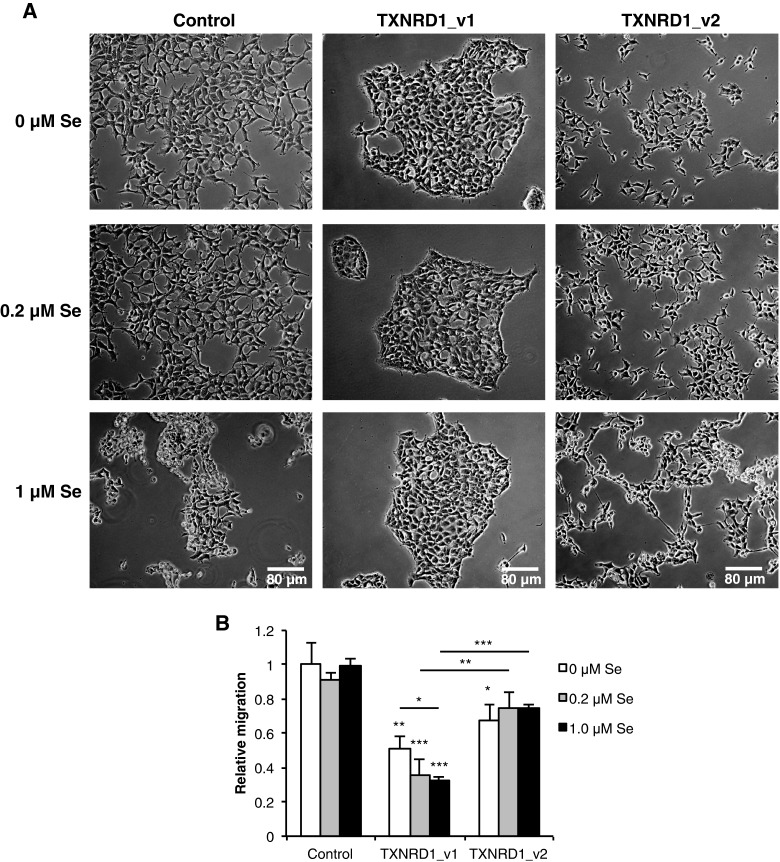
Morphological and migratory analysis of HEK-293 cells overexpressing TXNRD1v_1 and TXNRD1_v2 (**A**) Representative images of cells grown for 3 days in plain medium, 0.2 or 1 μM selenium-supplemented medium. Scale bar: 80 μm. (**B**) Cells grown for 3 days in 0, 0.2 or 1 μM Se were let to migrate for 24 h in Boyden chambers supplemented with 0, 0.2 or 1 μM Se. Error bars correspond to S.E.M. of three biological replicates, *p<0.05, **P<0.01, ***P<0.001.

### Gene expression analysis of HEK-293 cells overexpressing TXNRD1_v1 or TXNRD1_v2

Previously we reported on a more differentiated phenotype of HEK-293 cells overexpressing TXNRD1_v1 compared with control cells suggesting that this protein is an important player in differentiation processes [17]. This prompted us to analyse the genetic expression patterns in HEK-293 cells overexpressing TXNRD1_v1 or TXNRD1_v2 and explore whether the expression of genes associated with differentiation is altered in these cells compared with control HEK-293 cells transfected with the empty plasmid. Indeed, microarray analysis of these cells showed differential regulation of several genes not only associated with differentiation, but also with adhesion, cell polarity, migration, tumorigenesis and redox control (Table 1 and Supplementary Table I). Interestingly, although the HEK-TXNRD1_v2 cells resulted in a similar number of differentially expressed genes, the magnitude of change was lower and clustered closer to the control cells than to the HEK-TXNRD1_v1 cells (Supplementary Figure S2). As mentioned above, this effect may be linked to lower levels of TXNRD1_v2 and/or less selenium incorporation. Nevertheless, using the log odds cutoff *B* > 0 we could identify significant differential expression of 208 genes each in HEK-TXNRD1_v1 and TXNRD1_v2 cells compared with HEK-Control cells, of which 65 genes were shared between HEK-TXNRD1_v1 and TXNRD1_v2 cells (Table 1, Supplementary Figure S3, Supplementary Table I). High differences in expression levels was found for, among other, cadherin 11, galectin 8 and fibroblast growth factor 13, all involved in cell adhesion and/or differentiation (Table 1 and Supplementary Table I). Among the down-regulated genes we found SOX3 [SRY(sex determining region Y]-box 3) and oestrogen-related receptor γ (ERRγ), both having a role in differentiation and development of tissues. ERRγ, and two other genes encoding the selenoproteins GPx3 and selenoprotein T (SELT) were down-regulated only in TXNRD1_v1 overexpressing cells. These data were validated by quantitative real-time qPCR analysis (Table 1) and strongly suggest a role for both TXNRD1_v1 and TXNRD1_v2 in cell migratory and developmental processes. This was further strengthened when performing pathway analysis on the microarray data of HEK-TXNRD1_v1 overexpressing cells where the main affected pathways involve development, locomotion and redox control. (Supplementary Figure S2 and Supplementary Table I). Interestingly, fewer genes involved in redox homoeostasis were changed in the cells overexpressing TXNRD1_v2 (Supplementary Table I).

### TXNRD1_v1 and TXNRD1_v2 are overexpressed in differentiating SH-SY5Y neuroblastoma cells

The expression of genes associated with differentiation and adhesion in TXNRD1_v1/v2 overexpressing HEK-293 cells lead us to investigate whether induction of differentiation could increase the expression of TXNRD1_v1 and/or TXNRD1_v2. We made use of an established differentiation system, the neuroblastoma cell line (SH-SY5Y) treated with 5 μM ATRA [27]. SH-SY5Y cells are normally rounded in shape with short protrusions (Figure 4A). After 5 days of ATRA treatment the cells are morphologically fully differentiated and display a significant increase in characteristic long neuritic protrusions (Figure 4A). When analysing the protein levels of TXNRD1_v1/v2 by Western blot we could see an increase in both TXNRD1_v1 and TXNRD1_v2 protein levels in the fully differentiated cells (Figure 4B). Furthermore, five of the differentially expressed genes in the HEK-TXNRD1_v1/v2 cells (Supplementary Table I) were up-regulated in the ATRA treated SH-SY5Y cells by qPCR (Figure 4C); fibronectin 1, an adhesion glycoprotein; SOX9, normally expressed during gonadal development where it modulates proliferation and apoptosis but it also participates in programming liver and pancreatic progenitors [28]; cadherin 11, a tight junction trans membrane protein; ERRγ, a NR found in developing brain; and the adhesion molecule galectin 8.

**Figure 4:**
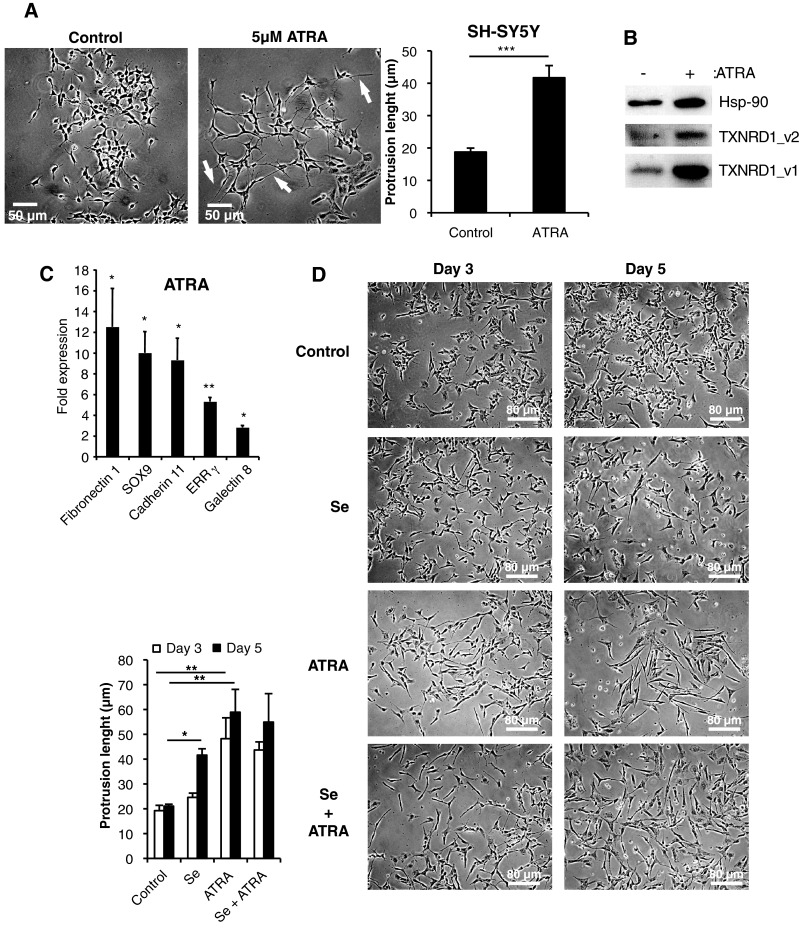
Differentiation of SH-SY5Y neuroblastoma cells (**A**) phase-contrast image of SH-SY5Y cell morphology after growth with or without 5 μM ATRA for 5 days. Characteristic neuritic morphology can be detected in differentiated cells by polar cell shape and neuritic protrusions (arrows). Right panel, quantification of protrusion length. Scale bar: 50 μm. (**B**) Western blot analysis of TXNRD1_v1 and TXNRD1_v2 in SH-SY5Y cells treated with or without 5 μM ATRA for 5 days. Hsp-90 was used as a loading control. (**C**), Real-time qPCR analysis of differentiated (5 days of 5 μM ATRA treatment) SH-SY5Y cells looking at genes found in TXNRD1_v1/v2 overexpressing cells (see Supplementary Table I). Values represent mean fold changes compared with untreated cells from at least three independent experiments completed in duplicates. (**D**) Phase-contrast image of SH-SY5Y cell morphology upon growth in plain medium, 0.2 μM Se, 5 μM ATRA or both selenium (3 days pretreated) and ATRA supplemented medium. Representative images were collected at days 3 and 5 of treatment. Bottom left panel, quantification of protrusion length. Scale bar: 80 μm. Error bars correspond to S.D., *P<0.05, **P<0.01, ***P<0.001.

Analysing the morphology of SH-SY5Y cells under selenium and/or ATRA treatment showed that cells already start to display a clear differentiated morphology at 3 days of 5 μM ATRA treatment (Figure 4D). Interestingly, incubating the cells with 0.2 μM Se for at least 5 days gave a clear change towards differentiated morphology compared with the untreated cells. However, combined selenium and ATRA supplementation did not increase short- or long-term morphology changes further compared with ATRA treatment alone (Figure 4D).

### TXNRD1_v1 and TXNRD1_v2 expression and activity affect expression of the adhesion molecules fibronectin 1 and cadherin 11 in differentiating SH-SY5Y cells

Upon treatment of the SH-SY5Y cells with 0.2 μM Se or 5 μM ATRA, the total TrxR activity was increased (Figure 5A). The selenium treatment caused a strong increase in TrxR activity at day 2 of selenium supplementation and was sustained until day 6 but dropped drastically thereafter. Treating the cells with ATRA increased the TrxR activity already at day 1 (Figure 5B) suggesting that differentiation of SH-SY5Y causes an elevated TrxR activity, however, this activity was not as strong as the selenium treatment alone. Treating the cells first with selenium for 3 days and thereafter combined selenium and ATRA treatment revealed a further small increase in TrxR activity on the first day of the combined treatment (Figure 5A), which, surprisingly, drastically decreased near to basal level thereafter.

**Figure 5:**
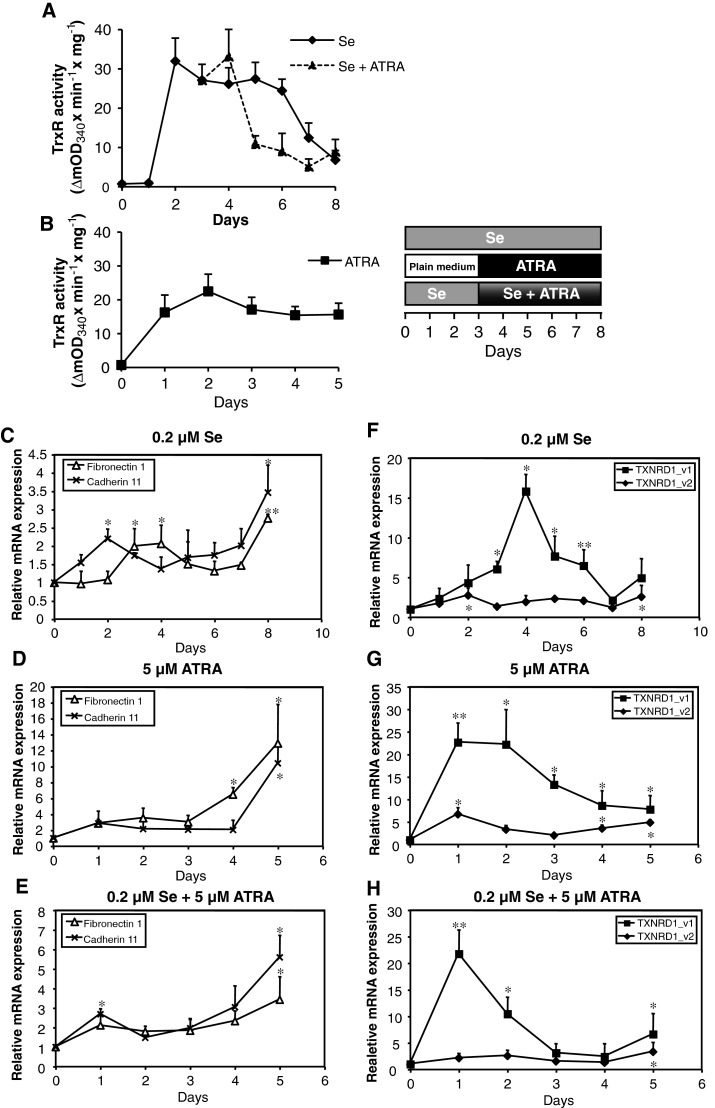
TrxR activity and expression in SH-SY5Y cells upon selenium and/or ATRA treatment: effect on fibronectin 1 and cadherin 11 expression (**A**) Total TrxR1 activity in SH-SY5Y cells measured by NADPH oxidation. The cells were pretreated (Se, Se + ATRA) with 0.2 μM Se for 3 days, and thereafter continued treated with 0.2 μM Se, 0.2 μM Se and 5 μM ATRA (Se + ATRA) or (**B**) cells were cultured for 3 days (without 0.2 μM Se) and then treated with 5 μM ATRA alone (ATRA). Right panel: experimental design. Real-time qPCR analysis of fibronectin 1, cadherin 11 TXNRD1_v1 and TXNRD1_v2 mRNA expression in SH-SY5Y cells with 0.2 μM Se **(C, F)**, 5 μM ATRA **(D, G)** or both 0.2 μM selenite (including 3 days selenite pretreatment) and 5 μM ATRA **(E, H)**, for indicated number of days. Real-time qPCR values represent mean fold changes compared with untreated cells from at least three independent experiments completed in duplicates. Error bars correspond to S.D., *P<0.05, **P<0.01.

Of the genes in Figure 4(C) found increased in the SH-SY5Y cells upon ATRA differentiation we saw a significant change in mRNA expression for two of those genes upon 0.2 μM selenium supplementation using the same experimental design as in Figures 5(A)and 5(B); cadherin 11 and fibronectin 1 (Figure 5C). Both are membrane proteins involved in cell adhesion and migration where cadherin 11 mediates cell–cell contacts and fibronectin 1 promotes cell-matrix contact to allow migration, cell guidance and intraneuronal interactions [29–31]. In SH-SY5Y cells, cadherin 11 and fibronectin 1 mRNA levels were approximately 2-fold increased already at days 2 and 3 of selenium treatment, respectively (Figure 5C). ATRA treatment gave a faster increase in cadherin 11 and fibronectin 1 levels (day 1) (Figure 5D) compared with selenium treatment, whereas the combined selenium and ATRA treatment (including 3 day selenium pretreatment) did not differ significantly from ATRA treatment alone (Figure 5E). When analysing TXNRD1_v1 and TXNRD1_v2 mRNA levels (Figure 5F) we could observe a rapid increase in TXNRD1_v1 expression upon selenium treatment. Interestingly, ATRA treatment increased the expression of both isoforms (Figure 5G). 3-day selenium pretreatment followed by 5 day ATRA and selenium treatment also increased expression of both isoforms (Figure 5H). Generally, the change in expression of TXNRD1_v1 was higher than that of TXNRD1_v2.

When analysing protein levels of TXNRD1_v1 and TXNRD1_v2, we observed a clear rapid increase upon selenium treatment (Figures 6A and 6D). We also observed an increase in cadherin 11 protein levels, but decreased fibronectin 1 protein levels (in contrast with mRNA levels) at days 1–3 of selenium treatment. This could possibly link increased TrxR1 activity with decreased fibronectin 1 levels and thus the decreased migration observed in Figure 3B. At day 8 both fibronectin 1 and cadherin 11 levels were increased, probably due to cell confluency. As expected, ATRA treatment increased both cadherin 11 and fibronectin 1 levels (days 3–5) (Figures 6B and 6E). Also, TXNRD1_v1 and TXNRD1_v2 levels were drastically increased upon ATRA treatment alone (Figures 6B and 6E). However, TXNRD1_v2 returned to control levels at day 2 whereas the levels of TXNRD1_v1 remained high. Interestingly, the increase in TXNRD1_v1 and TXNRD1_v2 levels upon ATRA treatment appeared earlier than the increase in fibronectin 1 and cadherin 11. Treating selenium-pretreated SH-SY5Y cells with selenium and ATRA for 5 days (Figures 6C and 6F) had initially no drastic effects on fibronectin 1 and cadherin 11 levels. The decrease in fibronectin 1 levels after the first selenium treatment was sustained. However, at later time points both fibronectin 1 and cadherin 11 levels were increased (Figures 6C and 6F). Combined selenium and ATRA treatment also increased TXNRD1_v1 and v2 levels, although we could not observe any additive effects. The opposite effect of selenium on fibronectin 1 and cadherin 11 protein levels suggests that TrxR1 activity to be important in keeping cell–cell contacts but not cell-matrix contacts during differentiation. The fast changes in TXNRD1_v2 protein levels compared with the more subtle changes in TXNRD1_v1 protein levels could imply different roles of TXNRD1_v1 and v2 on the cellular response to selenium and ATRA, the expression of fibronectin 1 and cadherin 11, and thus to migration and differentiation.

**Figure 6:**
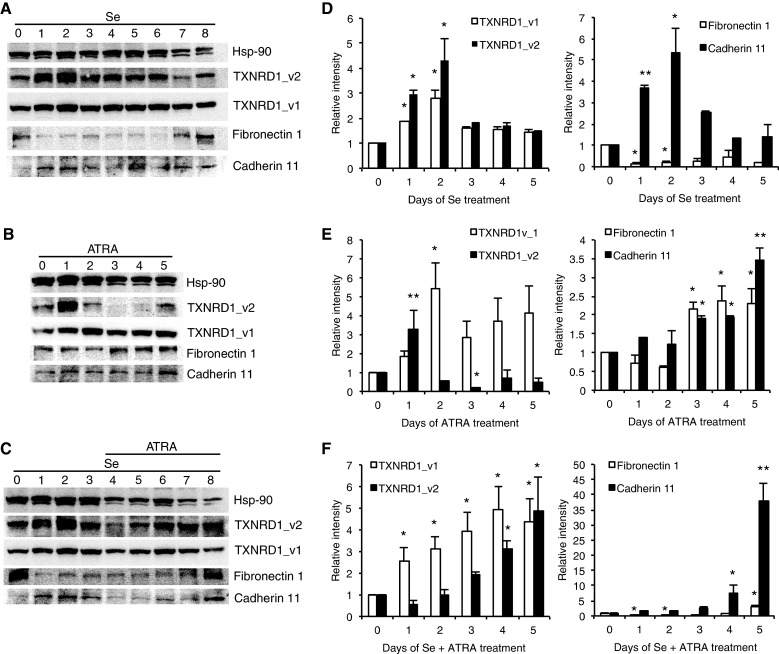
Comparison between TrxR, fibronectin 1 and cadherin 11 protein levels in SH-SY5Y cells upon selenium and/or ATRA treatment **(A**) Western blot analysis of TXNRD1_v1, TXNRD1_v2, fibronectin 1 and cadherin 11 in SH-SY5Y cell extracts upon indicated days of 0.2 μM Se, (**B**) 5 μM ATRA or (**C**) 0.2 μM Se and 5 μM ATRA treatment. (**D, E, F**) Left panels show quantified relative protein levels of TXNRD1_v1 and TXNRD1_v2 normalized to Hsp-90 levels for 5 days of respective treatment. Right panels show quantified relative protein levels of fibronectin 1 and Cadherin 11 normalized to Hsp-90 levels with the same treatment. Error bars correspond to S.D., *P<0.05, **P<0.01.

In summary, our results suggest that TXNRD1_v1 and TXNRD1_v2 are overexpressed in early stages of the differentiation process, and that they have different roles in regulating genes associated with differentiation and cell migration.

## DISCUSSION

Although the processes that initiate and regulate differentiation have been studied for many years, they are to a very large extent still unknown. However, it is known that the redox status in the cell is altered during different stages of cellular differentiation [32] and that several transcription factors associated with differentiation are known to be directly redox regulated [19,33,34]. TrxRs are potent mediators of the redox homoeostasis in the cell through the interaction with redox regulated molecules [35]. An altered redox balance, either fine-tuned and local or on a whole cellular scale, mediates induction of apoptosis, growth factor signalling events and an altered proliferation [36,37]. In the present study, we demonstrate that HEK-293 cells overexpressing either TXNRD1_v1 or its alternative splice variant TXNRD1_v2 express genes that, are not only involved in redox homoeostasis, but also affect pathways of development, differentiation and migration, proposing an unknown, but yet important, role for TrxRs in differentiation. To analyse whether TrxR expression is altered in differentiating cells we let the easily differentiating neuroblastoma cell line SH-SY5Y [38] differentiate towards a more neuritic phenotype by ATRA treatment. Apart from induced TXNRD1_v1/v2 expression, we confirmed seven more genes to be similarly expressed to the HEK overexpressing cells. Treating cells with selenium rapidly increases TrxR activity [17] and in doing so, we could observe altered expression of two of the above-analysed genes; cadherin 11 and fibronectin 1. Both cadherin 11 and fibronectin 1 are expressed at the cell surface as adhesion molecules. Cadherin 11 is known to mediate tight-junction formation and plays an important role in development, tissue architecture and modulation of cell migration [30,39,40]. Fibronectin 1 is known to be important for mediating the initiation of cell migration, and is also involved in axonal formation in neuronal development [29,31,41,42]. In HEK-293 cells TXNRD1_v1 and v2 overexpression increased the expresssion of cadherin 11, whereas only TXNRD1_v2 overexpression increased fibronectin 1 mRNA (Supplementary Table I) suggesting different biological functions of these splice variants. We could also observe a distinct cell morphology upon TXNRD1_v1 overexpression in HEK-293 cells, with tightly packed colonies, thus implying more cell–cell contacts and increased cadherin 11 expression, resulting in reduced cell motility. In fact it has been shown that the HEK-293 cells overexpressing TXNRD1_v1 used in the present study are less motile than control cells when stimulated [18]. We show that this is also the case in basal culturing conditions and that TXNRD1_v2 overexpression had less profound effects on migration with or without selenium stimulation than TXNRD1_v1 overexpression. The fact that fibronectin 1 is only increased in HEK-TXNRD1_v2 cells could possibly underlie the distinct features between TXNRD1_v2 and TXNRD1_v1 regarding cell migration.

Previously, Gorreta et al. [43] analysed the gene expression upon knockdown of TrxR1 by RNA interference (siRNA) in the hepatocellular carcinoma cell line HepG2. Interestingly, they also found several genes associated with cell adhesion, morphology, migration and differentiation to be differentially regulated upon TrxR1 gene silencing. Among those they found fibronectin 1. However, in contrast with our qPCR results, they found a slight overexpression of fibronectin 1 in their TrxR1 silencing experiments. It should, however, be taken into consideration that siRNA treatment down-regulated both the TXNRD1_v1 and TXNRD1_v2 genes. Evidently, these two splice forms seem to have different effects on the regulation of different genes. We saw that fibronectin 1 was up-regulated only upon TXNRD1_v2 overexpression, whereas TXNRD1_v1 had no effect (or a slight down-regulation). This further emphasizes the importance in distinguishing the actions between different TrxR1 splice forms, especially when studying the gene silencing or knockout of TrxR1 [34]. Cell line specific effects can also explain the differences between the published data and our study.

Although TXNRD1_v1 and its splice variant TXNRD1_v2 are mainly cytoplasmic proteins they are also found in the nucleus. TXNRD1_v2, which carries a LXXLL motif (a NR-box) known to bind NRs, has been reported to directly interact with both ERα and ERβ, modulating at least the transcriptional activity of ERβ [15,20]. Whether this interaction or any other interactions with NRs may result in a more differentiated cellular phenotype remains unclear. In our study, we could not see any significant change in nuclear/cytoplasmic localization of either TXNRD1_v1 or TXNRD1_v2 upon selenium supplementation (data not shown) suggesting that any nuclear effects of these are not dependent on their increased nuclear shuttling. However, in the present study we provide evidence that TXNRD1_v2 regulates some genes differently than TXNRD1_v1, which could be attributed to the difference in N-terminal structure. The expression of TXNRD1_v2 is lower than that of TXNRD1_v1, nevertheless we see that some genes are more up- or down-regulated by TXNRD1_v2 than TXNRD1_v1 in HEK-293 cells (Table 1 and Supplementary Table I). These genes include among other, fibronectin 1 and ERRγ. Interestingly, ERRγ is a NR, which could indicate a more direct interaction of TXNRD1_v2 with ERRγ or other NRs that may control its expression. Notably, ERRγ is expressed in a very tissue-specific manner during development and differentiation of tissues such as brain, kidney, liver, cardiac and skeletal muscle [44]. However, a clear function of ERRγ in differentiation is still not well characterized and further studies are necessary to elucidate the functions of this protein and its possible interactions with TrxRs. In addition to TXNRD1_v2, two other isoforms of TrxR1 have lately been shown to have NR-boxes in their alternative N-terminal domains; TGR [14] and TrxR-Grx (TXNRD1_v3) [45], further suggesting a role of TrxRs in NR signalling.

Our data support a role for TrxR in the early stages of differentiation possibly regulating cell adhesion and migration. This process could involve redox sensitive proteins such as transcription factors. In fact, as mentioned above, it is known that the activity of several redox sensitive transcription factors is regulated by the Trx system. Such transcription factors exhibit redox sensitive thiols in their DNA-binding motifs and TrxR1 has been shown to directly modulate the DNA-binding activity of at least oestrogen receptor α (ERα) [19], tumour suppressor protein p53 [46], hypoxia-inducible factor (HIF) [47], nuclear factor-kappa B (NF-κB) [48] and activator protein-1 (AP-1) [49,50]. Future studies will describe the different roles of TXNRD1_v1 and TXNRD1_v2 in the present study.

The main substrate of TrxR1 is Trx1. Nevertheless, the broad substrate specificity of TrxR1 plays important physiological roles. In fact, upon limited selenium supply a hierarchy of selenoprotein synthesis exists in the cell where TXNRD1_v1 is preferably synthesized over most other selenoproteins [17,51,52]. We can also observe this in our TXNRD1_v1 overexpressing cells where we see a slight, but significant, down-regulation of two other selenoproteins mRNA expression; GPx3 and SELT, where GPx3 is known to be a major acceptor of selenium in the organism. TrxR1 is also the main provider of the active form of selenium, selenide, for its own and all other selenoprotein synthesis by reducing selenium compounds, thus acting as a key enzyme in the selenium metabolism [53]. In addition, knockout of the selenium incorporation mechanism is embryonically lethal, implying a role for selenoproteins in development [54]. Although selenium in its low molecular mass compound forms of selenite, selenide, selenate and selenoamino acids are rather reactive compounds that could react with redox sensitive amino acids directly or at higher concentrations yield cell death, the biological effect of selenium, including cell signalling, differentiation, cell growth and survival, is still debated. However, its effect is considered to be primarily demonstrated through selenoproteins [55]. Notably, disrupting the TrxR1 or Trx1 genes has also shown to be lethal in mice, further proposing a role for the Trx-system in eukaryotic development [56,57]. However the individual TXNRD1_v1 or TXNRD1_v2 gene disruptions have not been characterized yet. In addition to affecting cellular differentiation pathways, TXNRD1_v1 overexpression decreased antioxidant pathways (Supplementary Table I), suggesting that TXNRD1_v1 overexpression affects the redox balance in the cell. This was not seen as evident with TXNRD1_v2 overexpressing cells, which have a phenotype more similar to control cells and clusters more with these cells than with TXNRD1_v1 overexpressing cells in the microarray analysis (Supplementary Figure S2).

## CONCLUSIONS

We show in the present study that the TxrR1 splice variants TXNRD1_v1 and TXNRD1_v2 are highly expressed and activated at an early stage of cellular differentiation, and that selenium treatment can induce differentiation phenotype in SH-SY5Y presumably through TrxR1 activation. Although the levels of both TXNRD1_v1 and v2 are increased in the differentiating cells, they seem to regulate expression of different adhesion molecules where TXNRD1_v1 could be more important for keeping cell–cell contacts and TXNRD1_v2 be more important for enabling cell migration. These findings together with previously published data showing that TrxR1s are indispensable in embryogenesis [56] and involved in the regulation of genes associated with differentiation and migration [17,18], put forward an important role for TrxR1s in the developmental processes.

## Abbreviations

ATRA: all-*trans* retinoic acid
DMEM: Dulbecco's-modified Eagle's medium
ER: oestrogen receptor
ERRγ: oestrogen-related receptor γ
GPx: glutathione peroxidase
HEK-293: human embryonic kidney cell line
NR: nuclear receptor
PEI: polyethyleneimine
Se: sodium selenite
SELT: selenoprotein T
SOX: SRY(sex determining region Y)-box
Trx: thioredoxin
TrxR: thioredoxin reductase

## References

[1] Holmgren A. (1989). Thioredoxin and glutaredoxin systems. J. Biol. Chem..

[2] Arner E.S., Holmgren A. (2000). Physiological functions of thioredoxin and thioredoxin reductase. Eur. J. Biochem..

[3] Lu J., Berndt C., Holmgren A. (2009). Metabolism of selenium compounds catalyzed by the mammalian selenoprotein thioredoxin reductase. Biochim. Biophys. Acta.

[4] Mahmood D.F., Abderrazak A., El Hadri K., Simmet T., Rouis M. (2013). The thioredoxin system as a therapeutic target in human health and disease. Antioxid. Redox Signal..

[5] Mustacich D., Powis G. (2000). Thioredoxin reductase. Biochem. J..

[6] Gladyshev V.N., Jeang K.T., Stadtman T.C. (1996). Selenocysteine, identified as the penultimate C-terminal residue in human T-cell thioredoxin reductase, corresponds to TGA in the human placental gene. Proc. Natl. Acad. Sci. U.S.A..

[7] Zhong L., Arner E.S., Holmgren A. (2000). Structure and mechanism of mammalian thioredoxin reductase: the active site is a redox-active selenolthiol/selenenylsulfide formed from the conserved cysteine-selenocysteine sequence. Proc. Natl. Acad. Sci. U.S.A..

[8] Sandalova T., Zhong L., Lindqvist Y., Holmgren A., Schneider G. (2001). Three-dimensional structure of a mammalian thioredoxin reductase: implications for mechanism and evolution of a selenocysteine-dependent enzyme. Proc. Natl. Acad. Sci. U.S.A..

[9] Nordberg J., Arner E.S. (2001). Reactive oxygen species, antioxidants, and the mammalian thioredoxin system. Free Radic. Biol. Med..

[10] Arner E.S. (2009). Focus on mammalian thioredoxin reductases—important selenoproteins with versatile functions. Biochim. Biophys. Acta.

[11] Xia L., Nordman T., Olsson J.M., Damdimopoulos A., Bjorkhem-Bergman L., Nalvarte I., Eriksson L.C., Arner E.S., Spyrou G., Bjornstedt M. (2003). The mammalian cytosolic selenoenzyme thioredoxin reductase reduces ubiquinone. A novel mechanism for defense against oxidative stress. J. Biol. Chem..

[12] Miranda-Vizuete A., Damdimopoulos A.E., Pedrajas J.R., Gustafsson J.A., Spyrou G. (1999). Human mitochondrial thioredoxin reductase cDNA cloning, expression and genomic organization. Eur. J. Biochem..

[13] Miranda-Vizuete A., Damdimopoulos A.E., Spyrou G. (2000). The mitochondrial thioredoxin system. Antioxid. Redox Signal..

[14] Sun Q.A., Kirnarsky L., Sherman S., Gladyshev V.N. (2001). Selenoprotein oxidoreductase with specificity for thioredoxin and glutathione systems. Proc. Natl. Acad. Sci. U.S.A..

[15] Su D., Gladyshev V.N. (2004). Alternative splicing involving the thioredoxin reductase module in mammals: a glutaredoxin-containing thioredoxin reductase 1. Biochemistry.

[16] Rundlof A.K., Fernandes A.P., Selenius M., Babic M., Shariatgorji M., Nilsonne G., Ilag L.L., Dobra K., Bjornstedt M. (2007). Quantification of alternative mRNA species and identification of thioredoxin reductase 1 isoforms in human tumor cells. Differentiation.

[17] Nalvarte I., Damdimopoulos A.E., Nystom C., Nordman T., Miranda-Vizuete A., Olsson J.M., Eriksson L., Bjornstedt M., Arner E.S., Spyrou G. (2004). Overexpression of enzymatically active human cytosolic and mitochondrial thioredoxin reductase in HEK-293 cells. Effect on cell growth and differentiation. J. Biol. Chem..

[18] Sroka J., Antosik A., Czyz J., Nalvarte I., Olsson J.M., Spyrou G., Madeja Z. (2007). Overexpression of thioredoxin reductase 1 inhibits migration of HEK-293 cells. Biol. Cell.

[19] Rao A.K., Ziegler Y.S., McLeod I.X., Yates J.R., Nardulli A.M. (2009). Thioredoxin and thioredoxin reductase influence estrogen receptor alpha-mediated gene expression in human breast cancer cells. J. Mol. Endocrinol..

[20] Damdimopoulos A.E., Miranda-Vizuete A., Treuter E., Gustafsson J.A., Spyrou G. (2004). An alternative splicing variant of the selenoprotein thioredoxin reductase is a modulator of estrogen signaling. J. Biol. Chem..

[21] Holmgren A., Bjornstedt M. (1995). Thioredoxin and thioredoxin reductase. Methods Enzymol..

[22] Psarra A.M., Hermann S., Panayotou G., Spyrou G. (2009). Interaction of mitochondrial thioredoxin with glucocorticoid receptor and NF-kappaB modulates glucocorticoid receptor and NF-kappaB signalling in HEK-293 cells. Biochem. J..

[23] Miranda-Vizuete A., Spyrou G. (1997). The novel oxidoreductase KDRF (KM-102-derived reductase-like factor) is identical with human thioredoxin reductase. Biochem. J..

[24] Koishi R., Kawashima I., Yoshimura C., Sugawara M., Serizawa N. (1997). Cloning and characterization of a novel oxidoreductase KDRF from a human bone marrow-derived stromal cell line KM-102. J. Biol. Chem..

[25] Leist M., Raab B., Maurer S., Rosick U., Brigelius-Flohe R. (1996). Conventional cell culture media do not adequately supply cells with antioxidants and thus facilitate peroxide-induced genotoxicity. Free Radic. Biol. Med..

[26] Madeja Z., Sroka J., Nystrom C., Bjorkhem-Bergman L., Nordman T., Damdimopoulos A., Nalvarte I., Eriksson L.C., Spyrou G., Olsson J.M., Bjornstedt M. (2005). The role of thioredoxin reductase activity in selenium-induced cytotoxicity. Biochem. Pharmacol..

[27] Sallmon H., Hoene V., Weber S.C., Dame C. (2010). Differentiation of human SH-SY5Y neuroblastoma cells by all-*trans* retinoic acid activates the interleukin-18 system. J. Interferon Cytokine Res..

[28] Kawaguchi Y. (2013). Sox9 and programming of liver and pancreatic progenitors. J. Clin. Invest..

[29] Pankov R., Yamada K.M. (2002). Fibronectin at a glance. J. Cell Sci..

[30] Huang C.F., Lira C., Chu K., Bilen M.A., Lee Y.C., Ye X., Kim S.M., Ortiz A., Wu F.L., Logothetis C.J. (2010). Cadherin-11 increases migration and invasion of prostate cancer cells and enhances their interaction with osteoblasts. Cancer Res..

[31] Sheppard A.M., Brunstrom J.E., Thornton T.N., Gerfen R.W., Broekelmann T.J., McDonald J.A., Pearlman A.L. (1995). Neuronal production of fibronectin in the cerebral cortex during migration and layer formation is unique to specific cortical domains. Dev. Biol..

[32] Allen R.G., Newton R.K., Sohal R.S., Shipley G.L., Nations C. (1985). Alterations in superoxide dismutase, glutathione, and peroxides in the plasmodial slime mold Physarum polycephalum during differentiation. J. Cell. Physiol..

[33] Leonarduzzi G., Sottero B., Testa G., Biasi F., Poli G. (2011). New insights into redox-modulated cell signaling. Curr. Pharm. Des..

[34] Abate C., Patel L., Rauscher F.J., Curran T. (1990). Redox regulation of fos and jun DNA-binding activity *in vitro*. Science.

[35] Rundlof A.K., Arner E.S. (2004). Regulation of the mammalian selenoprotein thioredoxin reductase 1 in relation to cellular phenotype, growth, and signaling events. Antioxid. Redox Signal..

[36] Schreck R., Baeuerle P.A. (1991). A role for oxygen radicals as second messengers. Trends Cell Biol..

[37] Sundaresan M., Yu Z.X., Ferrans V.J., Irani K., Finkel T. (1995). Requirement for generation of H_2_O_2_ for platelet-derived growth factor signal transduction. Science.

[38] Pahlman S., Ruusala A.I., Abrahamsson L., Mattsson M.E., Esscher T. (1984). Retinoic acid-induced differentiation of cultured human neuroblastoma cells: a comparison with phorbolester-induced differentiation. Cell Differ..

[39] Richardson S.H., Starborg T., Lu Y., Humphries S.M., Meadows R.S., Kadler K.E. (2007). Tendon development requires regulation of cell condensation and cell shape via cadherin-11-mediated cell–cell junctions. Mol. Cell. Biol..

[40] Schulte J.D., Srikanth M., Das S., Zhang J., Lathia J.D., Yin L., Rich J.N., Olson E.C., Kessler J.A., Chenn A. (2013). Cadherin-11 regulates motility in normal cortical neural precursors and glioblastoma. PLoS One.

[41] Du Y.H., Hirooka K., Miyamoto O., Bao Y.Q., Zhang B., An J.B., Ma J.X. (2013). Retinoic acid suppresses the adhesion and migration of human retinal pigment epithelial cells. Exp. Eye Res..

[42] Pires Neto M.A., Braga-de-Souza S., Lent R. (1999). Extracellular matrix molecules play diverse roles in the growth and guidance of central nervous system axons. Braz. J. Med. Biol. Res..

[43] Gorreta F., Runfola T.P., VanMeter A.J., Barzaghi D., Chandhoke V., Del Giacco L. (2005). Identification of thioredoxin reductase 1-regulated genes using small interference RNA and cDNA microarray. Cancer Biol. Ther..

[44] Heard D.J., Norby P.L., Holloway J., Vissing H. (2000). Human ERRgamma, a third member of the estrogen receptor-related receptor (ERR) subfamily of orphan nuclear receptors: tissue-specific isoforms are expressed during development and in the adult. Mol. Endocrinol..

[45] Damdimopoulou P.E., Miranda-Vizuete A., Arner E.S., Gustafsson J.A., Damdimopoulos A.E. (2009). The human thioredoxin reductase-1 splice variant TXNRD1_v3 is an atypical inducer of cytoplasmic filaments and cell membrane filopodia. Biochim. Biophys. Acta.

[46] Cassidy P.B., Edes K., Nelson C.C., Parsawar K., Fitzpatrick F.A., Moos P.J. (2006). Thioredoxin reductase is required for the inactivation of tumor suppressor p53 and for apoptosis induced by endogenous electrophiles. Carcinogenesis.

[47] Moos P.J., Edes K., Cassidy P., Massuda E., Fitzpatrick F.A. (2003). Electrophilic prostaglandins and lipid aldehydes repress redox-sensitive transcription factors p53 and hypoxia-inducible factor by impairing the selenoprotein thioredoxin reductase. J. Biol. Chem..

[48] Matthews J.R., Wakasugi N., Virelizier J.L., Yodoi J., Hay R.T. (1992). Thioredoxin regulates the DNA binding activity of NF-kappa B by reduction of a disulphide bond involving cysteine 62. Nucleic Acids Res..

[49] Seemann S., Hainaut P. (2005). Roles of thioredoxin reductase 1 and APE/Ref-1 in the control of basal p53 stability and activity. Oncogene.

[50] Karimpour S., Lou J., Lin L.L., Rene L.M., Lagunas L., Ma X., Karra S., Bradbury C.M., Markovina S., Goswami P.C. (2002). Thioredoxin reductase regulates AP-1 activity as well as thioredoxin nuclear localization via active cysteines in response to ionizing radiation. Oncogene.

[51] Low S.C., Grundner-Culemann E., Harney J.W., Berry M.J. (2000). SECIS–SBP2 interactions dictate selenocysteine incorporation efficiency and selenoprotein hierarchy. EMBO J..

[52] Moustafa M.E., Carlson B.A., El-Saadani M.A., Kryukov G.V., Sun Q.A., Harney J.W., Hill K.E., Combs G.F., Feigenbaum L., Mansur D.B. (2001). Selective inhibition of selenocysteine tRNA maturation and selenoprotein synthesis in transgenic mice expressing isopentenyladenosine-deficient selenocysteine tRNA. Mol. Cell. Biol..

[53] Bjornstedt M., Odlander B., Kuprin S., Claesson H.E., Holmgren A. (1996). Selenite incubated with NADPH and mammalian thioredoxin reductase yields selenide, which inhibits lipoxygenase and changes the electron spin resonance spectrum of the active site iron. Biochemistry.

[54] Bosl M.R., Takaku K., Oshima M., Nishimura S., Taketo M.M. (1997). Early embryonic lethality caused by targeted disruption of the mouse selenocysteine tRNA gene (Trsp). Proc. Natl. Acad. Sci. U.S.A..

[55] McKenzie R.C., Arthur J.R., Beckett G.J. (2002). Selenium and the regulation of cell signaling, growth, and survival: molecular and mechanistic aspects. Antioxid. Redox Signal..

[56] Jakupoglu C., Przemeck G.K., Schneider M., Moreno S.G., Mayr N., Hatzopoulos A.K., de Angelis M.H., Wurst W., Bornkamm G.W., Brielmeier M., Conrad M. (2005). Cytoplasmic thioredoxin reductase is essential for embryogenesis but dispensable for cardiac development. Mol. Cell. Biol..

[57] Matsui M., Oshima M., Oshima H., Takaku K., Maruyama T., Yodoi J., Taketo M.M. (1996). Early embryonic lethality caused by targeted disruption of the mouse thioredoxin gene. Dev. Biol..

